# A comparison of different methods to handle missing data in the context of propensity score analysis

**DOI:** 10.1007/s10654-018-0447-z

**Published:** 2018-10-19

**Authors:** Jungyeon Choi, Olaf M. Dekkers, Saskia le Cessie

**Affiliations:** 1 Department of Clinical Epidemiology, Leiden University Medical Center, Albinusdreef 2, C7-P, 2333 ZA Leiden, The Netherlands; 2 Department of Endocrinology and Metabolism, Leiden University Medical Center, Albinusdreef 2, C7-P, 2333 ZA Leiden, The Netherlands; 3 Department of Biomedical Data Sciences, Leiden University Medical Center, Albinusdreef 2, C7-P, 2333 ZA Leiden, The Netherlands

**Keywords:** Missing data, Propensity score analysis, Multiple imputation, Missing indicator, Effect modification, Missingness graph

## Abstract

Propensity score analysis is a popular method to control for confounding in observational studies. A challenge in propensity methods is missing values in confounders. Several strategies for handling missing values exist, but guidance in choosing the best method is needed. In this simulation study, we compared four strategies of handling missing covariate values in propensity matching and propensity weighting. These methods include: complete case analysis, missing indicator method, multiple imputation and combining multiple imputation and missing indicator method. Concurrently, we aimed to provide guidance in choosing the optimal strategy. Simulated scenarios varied regarding missing mechanism, presence of effect modification or unmeasured confounding. Additionally, we demonstrated how missingness graphs help clarifying the missing structure. When no effect modification existed, complete case analysis yielded valid causal treatment effects even when data were missing not at random. In some situations, complete case analysis was also able to partially correct for unmeasured confounding. Multiple imputation worked well if the data were missing (completely) at random, and if the imputation model was correctly specified. In the presence of effect modification, more complex imputation models than default options of commonly used statistical software were required. Multiple imputation may fail when data are missing not at random. Here, combining multiple imputation and the missing indicator method reduced the bias as the missing indicator variable can be a proxy for unobserved confounding. The optimal way to handle missing values in covariates of propensity score models depends on the missing data structure and the presence of effect modification. When effect modification is present, default settings of imputation methods may yield biased results even if data are missing at random.

## Introduction

Observational studies potentially suffer from confounding. Propensity score methods, first introduced by Rosenbaum and Rubin [1], are increasingly being used in medical research to handle confounding [2–5]. When the observed baseline characteristics are sufficient to correct for confounding bias and the propensity model is correctly specified, propensity score analysis creates conditional exchangeability between persons with the same propensity score. Numerous studies provide illustrations and discussions on the performance of different propensity score approaches [3, 4, 6–11].

Besides confounding, observational studies often have missing values in covariates. Missing values can occur by different mechanisms: values are *missing completely at random* (MCAR) when the probability that a value is missing is independent from observed and unobserved information (e.g. a lab measurement is missing, because a technician dropped a tube), *missing at random* (MAR) where the probability of missing depends only on observed information (e.g. lab measurements are only performed when other measured variables were abnormal), or *missing not at random* (MNAR) where the probability of missing depends on unobserved information (e.g. lab measurements are only performed when a doctor judged that a patient was in a severe condition, while the severity is not well-registered.) [12]. However, it is difficult to decide on the type of missing mechanism, especially when distinguishing whether the data are *missing at random* or *not at random* [13, 14]. Especially in routinely collected data, variables are often selectively measured based on a patient’s characteristics which are often not well-specified [15]. If those ill-defined characteristics are associated with the variable with missing values, data is missing not at random. External knowledge or assumptions about the clinical setting are required to distinguish whether the missing is at random or not at random.

How to estimate propensity scores when there are missing values is a challenge when studying causal associations [16]. There are different strategies to handle missing data in a propensity score analysis. The simplest approach is to discard all observations with missing data, a so-called complete case analysis [12, 17]. Including a missing indicator in a statistical model is another simple method. However, various studies showed that the method in general introduce bias [18–21]. Multiple imputation is a standard method to deal with missing data. Many studies have shown the advantage of multiple imputation and its superiority over other methods [12, 19, 22]. In combination with propensity scores, however, several questions arise: Should we include the outcome in the imputation model? Can we use the imputation methods implemented in standard software? How should we combine the results of the different propensity scores estimated in each imputed dataset?

The aim of this simulation study is to investigate how different strategies of handling missing values of covariates in a propensity score model can yield valid causal treatment effect estimates. To limit the scope of the study, we deal only with missing values in the baseline characteristics, which is a rather common situation happens in routinely collected data. We create simulation scenarios varying in their missing data mechanisms, presence of heterogeneous treatment effect and unmeasured confounding. Subsequently, the results are used to provide guidance in choosing an optimal strategy to handle missing data in the context of propensity score analysis.

## Simulation description

We generated simulated data with missing values in one of the confounders and compared effect estimates obtained by using several different strategies to deal with missing data. In Sect. 2.1 we considered a situation without unmeasured confounding and with the equal treatment effect for all subjects. In Sect. 2.2, we introduced a heterogeneous treatment effect. In Sect. 2.3, the simulations were extended by adding unmeasured confounding.

### Simulation setting 1: No unmeasured confounding and a homogeneous treatment effect

In this simulation series, for each subject we generated two continuous covariates X_1_ and X_2_. X_1_ follows a normal distribution of mean 0 and standard deviation of 1. X_2_ depended on X_1_, where for subject *i*,

$$X_{2i} = 0.5 X_{1i} + \varepsilon_{i} \quad {\text{with}} \;\varepsilon_{i} \,\sim\,N\left( {0, 0.75} \right)$$

In this way the standard deviation of X_2_ is also 1 and the correlation between X_1_ and X_2_ is equal to 0.5. The treatment T was generated from the binomial distribution, with the probability for subject *i* to receive the treatment being equal to:

$$\begin{array}{*{20}c} {logit\left( {P\left( {T_{i} = 1 | {\text{X}}_{1i} , {\text{X}}_{2i} } \right)} \right) = - 0.8 + 0.5 {\text{X}}_{1i} + 0.5 {\text{X}}_{2i} } \\ \end{array}$$

In this way about 33% of the generated subjects received treatment. A continuous outcome was generated with the mean linearly related to X_1_ and X_2_:

$$\begin{array}{*{20}c} {{\text{Y}}_{i} = {\text{X}}_{1i} + {\text{X}}_{2i} + \varepsilon_{i} ,\quad {\text{with}}\; \varepsilon_{i} \,\sim\,N\left( {0, 1} \right)} \\ \end{array}$$

For ease of interpretation of the results, we assumed that the treatment T had no effect on the outcome for any of the subjects. Missing data were generated for 50% of the X_2_ values in three different ways:

- A missing completely at random (MCAR) scenario: 50% of values are randomly set to missing in X_2._
- A missing at random (MAR) scenario: The higher the value of X_1_, the more likely for the X_2_ value to be missing. Denoting R as a missing indicator of X_2_, the probability of a missing X_2_ value was equal to:
  $$\begin{array}{*{20}c} {logit\left( {P\left( {R_{i} = 1} \right)} \right) = {\text{X}}_{1i} } \\ \end{array}$$
- A missing not at random (MNAR) scenario: The higher the value of X_2_, the more likely that the value was missing. The probability of a missing X_2_ value was:
  $$\begin{array}{*{20}c} {logit\left( {P\left( {R_{i} = 1} \right)} \right) = {\text{X}}_{2i} } \\ \end{array}$$

**Fig. 1:**
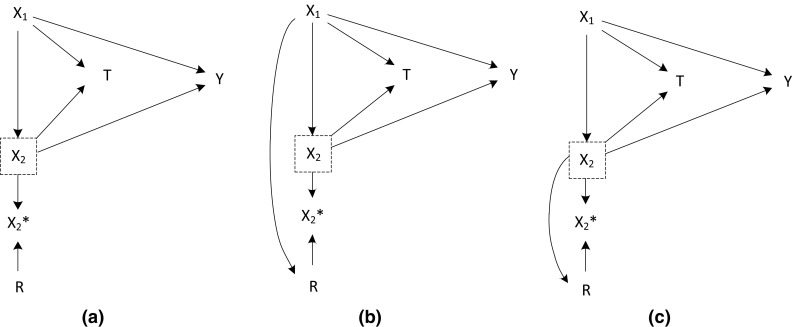
M-graphs for Simulation setting 1: MCAR scenario (**a**), MAR scenario (**b**), and MANR scenario (**c**)

Missingness-graphs (*m*-graph, for short) of each missing scenario are depicted in Fig. 1. The missingness graph is a graphical tool to represent missing data, proposed by Mohan et al. [23]. Guidance for practical users is given in Thoemmes and Mohan [24]. These graphs are extensions to causal directed acyclic graphs (DAGs) where nodes indicate covariates and arrows indicate causal relations. When a covariate contains missing values (X_2_ in our simulations), it is expressed by a dashed rectangle around the node. The node R represents the missingess of X_2_, and can be referred to as a missing indicator of X_2_. The observed portion of X_2_ is represented as X_2_*. When R = 0, X_2_* is identical to X_2_, and when R = 1, X_2_* is missing. In our simulations we restricted ourselves to the situation where missing values occur only in one covariate. However, *m*-graphs can be extended to situations with multiple covariates having missing values and, accordingly, with multiple missing indicator variables.

### Simulation setting 2: No unmeasured confounding and a heterogeneous treatment effect

The setup of this simulation series is the same as in Simulation setting 1, but here we assumed effect modification by X_2_. That is,

$$\begin{array}{*{20}c} {{\text{Y}}_{i} = {\text{X}}_{1i} + {\text{X}}_{2i} + {\text{T}}_{i} {\text{X}}_{2i} + \varepsilon_{i} ,\quad {\text{with}}\; \varepsilon_{i} \,\sim\,N\left( {0,1} \right)} \\ \end{array}$$

**Fig. 2:**
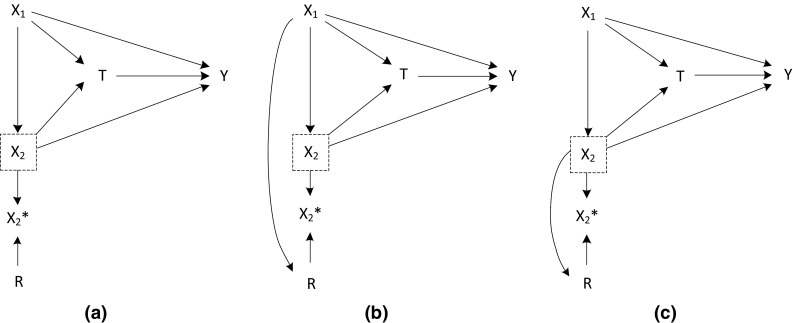
M-graphs for Simulation setting 2: MCAR scenario (**a**), MAR scenario (**b**), and MANR scenario (**c**)

The average treatment effect in the population was equal to null as in Simulation setting 1. However, due to the effect modification by X_2_, the average treatment effect was negative for subjects with X_2_ < 0 and positive for subjects with X_2_ > 0. Missing values were generated in the X_2_ variable, following the same mechanisms as in Simulation setting 1. The *m*-graphs for each scenario are depicted in Fig. 2. In these m-graphs, there is an arrow from the treatment assignment (T) to the outcome (Y), because for some subjects the treatment has an effect on their outcome.

### Simulation setting 3: Unmeasured confounding and a homogeneous treatment effect

In this series of simulations, we assumed an additional unobserved confounder U, normally distributed with a mean of 0 and standard deviation of 1 and independent from X_1_. X_2_ depended on X_1_ and U, where for subject *i*,

$${\text{X}}_{2i} = 0.5 {\text{X}}_{1i} + 0.5 {\text{U}}_{i} + \varepsilon_{i} \quad {\text{with}}\; \varepsilon_{i} \,\sim\,N\left( {0, 0.5} \right)$$

The probability of receiving the treatment depended on X_1_, X_2_ and U as follows:

$$\begin{array}{*{20}c} {logit\left( {P\left( {T_{i} = 1 | {\text{X}}_{1i} , {\text{X}}_{2i} ,{\text{U}}_{i} } \right)} \right) = - 0.85 + 0.5 {\text{X}}_{1i} + 0.5 {\text{X}}_{2i} + 0.5 {\text{U}}_{i} } \\ \end{array}$$

In this way about 33% of the generated subjects received the treatment. The outcome now depended on X_1_, X_2_ and U:

$$\begin{array}{*{20}c} {{\text{Y}}_{i} = {\text{X}}_{1i} + {\text{X}}_{2i} + {\text{U}}_{i} + \varepsilon_{i} , \quad {\text{with }}\;\varepsilon_{i} \,\sim\,N\left( {0,1} \right)} \\ \end{array}$$

Here, we assumed a homogeneous treatment effect which was set to null. We considered two missing scenarios for X_2_; one according to the MCAR mechanism and the other MNAR mechanism.

- A MCAR scenario: 50% of values are randomly set to be missing in X_2._
- A MNAR scenario: Here we considered a common situation in routinely collected health care data where the missing of X_2_ depended on the unobserved confounder U. We set the value of X_2_ to be missing if U > 0.

**Fig. 3:**
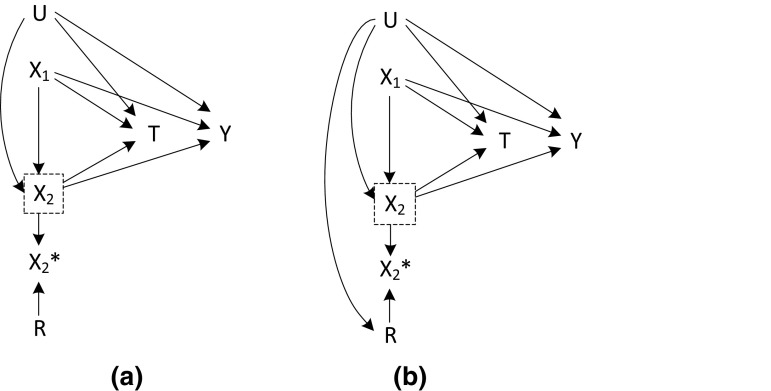
M-graphs for Simulation setting 3: MCAR scenario (**a**), MNAR scenario (**b**)

A MAR scenario was not considered in this simulation setting. This is because we were interested in comparing a situation where an unmeasured confounder U affect the missingness of X_2_ (MNAR) to a situation where it does not affect the missingness of X_2_ (MCAR). The m-graphs for these scenarios are illustrated in Fig. 3.

### Analysis of the simulated datasets

In every simulated dataset, we estimated propensity scores by logistic regression. The treatment effect was estimated by (1) propensity matching and (2) propensity weighting. For the matching procedure, we matched a treated subject to an untreated subject by using one-to-one nearest neighbour matching without replacement and 0.1 caliper distance on the logit scale. In the propensity weighting, the so-called inverse probability weighting, treated subjects are weighted by *1/propensity score*, and untreated subjects are weighted by *1/(1 *− *propensity score)*. Note that causal effects estimated by propensity matching and propensity weighting are different from each other. The matching estimates the average treatment effect in the *treated population*, while the weighting method estimates the average treatment effect in the *total population*. For handling missing values, we applied following four different methods.

#### Complete case analysis

In this approach, only observations with complete information are used for analysis.

#### Missing indicator method

When a covariate contains missing values, they were replaced by one single value, for example by the value 0. Additionally, a missing indicator variable was created with the value 1 indicating that the corresponding value is missing and the value 0 indicating that it is observed. The missing indicator variable was then added as a covariate in a propensity score model. When there are multiple covariates with missing values, missing indicators will be created for each covariate which will be all added to a propensity score model.

#### Multiple imputation

The third method considered was multiple imputation. Here the chained equation (MICE) procedure, a commonly used imputation method that assumes data are missing at random [25], was used. We used the default options of MICE version 3.3.0 [26] in R version 3.5.1: predictive mean matching via a regression model with main effects of X_1_, X_2_, T and with or without Y. In this way, the simulations reflect how most applied researchers using R would perform multiple imputation. Predictive mean matching is also readily available in SAS version 9.4, Stata version 15 and IBM SPSS version 25.0, and it is recommended when data contains both continuous and discrete values [27, 28]. As a sensitivity analysis we repeated Simulation setting 2 by using MICE with Bayesian linear regression, since many researchers will opt for this method when covariates and outcomes are continuous.

In Simulation setting 2, where a heterogeneous treatment effect exists, we additionally used a more extensive imputation model with three interaction terms included; the interaction between T and X_1_, T and Y, and X_1_ and Y. Adding interaction terms between the variables in an multiple imputation regression model is advocated by Tilling et al. [29]. For every multiple imputation, ten imputed datasets were generated. A treatment effect was estimated within each imputed dataset using the propensity score methods. Using Rubin’s rule, the ten treatment effects were then combined into a single treatment effect. This method is referred to as the within method [30].

We explored whether the outcome should be included in the imputation model. The idea behind the propensity score methods is that the probability of receiving the treatment is modelled without knowing the outcome [16], which is why some researchers argue that the outcome should not be used in the imputation model [31]. The purpose of multiple imputation, however, is a reconstruction of data to retain the original relationship between the covariates as much as possible, for which the outcome could provide valuable information [32–35]. This suggests that the outcome should be added in an imputation model.

#### Multiple imputation together with missing indicator

The fourth method was a combination of multiple imputation and the missing indicator method. Multiple imputation was used to impute the missing values. Afterward, both the imputed covariate and a missing indicator variable were added in the propensity score model [36]. Multiple imputation was performed following the same procedure as in Sect. 2.4.3, where the treatment effect is estimated by the within method.

### Simulation summary

Each simulation run generated a thousand observations and was repeated for a thousand times. We summarised the simulation results by calculating the mean treatment effects over the simulations and the standard deviation of the estimated treatment effects. As overall measures of performance, we calculated the mean squared error, which is the squared distance between the estimated treatment effect and the true treatment effect averaged over the simulations.

In Simulation setting 1 and 3, the true treatment effect was null for all subjects, which means that the mean estimated treatment effects deviating from 0 demonstrates bias has been introduced. In Simulation setting 2, the average treatment effect in the *population*; the causal effect estimated by propensity weighting, was also equal to null. However, due to the heterogeneous treatment effect, the average treatment effect in the *treated*; the causal effect estimated by propensity matching, differed from null. In this simulation setting, the treatment effect for individual *i* is equal to X_2*i*_ which implies the average treatment effect in the treated would be E[X_2_|T = 1]. In this simulated example E[X_2_|T = 1] was equal to 0.432.

## Results

### Simulation setting 1: No unmeasured confounding and a homogeneous treatment effect

**Fig. 4:**
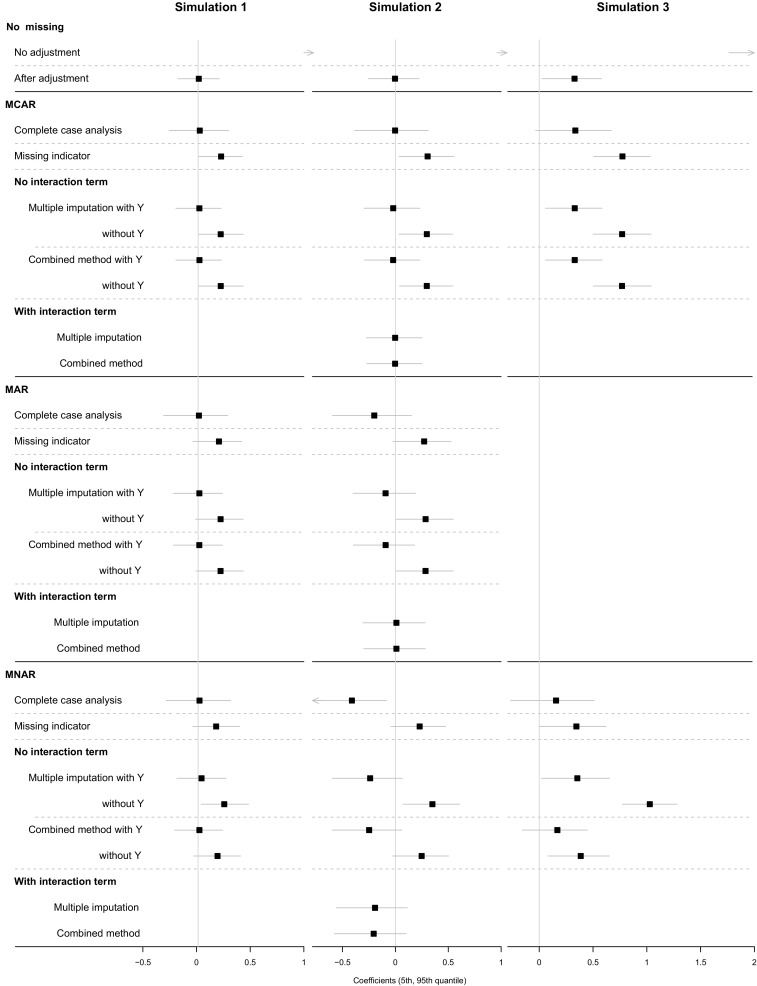
Mean treatment effects and their 5th and 95th percentile ranges estimated by propensity weighting in Simulation setting 1 (left), 2 (middle) and 3 (right). For each missing scenario, missing data are handled with complete case analysis, missing indicator method, multiple imputation, and the combination of multiple imputation and missing indicator method (Combined method). The vertical lines represent the true treatment effect

Figure 4 (left) displays the mean estimated effects of the propensity weighting analysis in Simulation setting 1 and their 5th and 95th percentile range. Table 1 shows the mean estimates with standard deviations and mean squared errors from the propensity matching and the propensity weighting. Complete case analysis yielded unbiased treatment effect estimates in all scenarios, even when data were missing not at random. The missing indicator method alone resulted in biased estimates in all scenarios. The results suggested that the outcome should be included in an imputation model, since the imputation models not including the outcome resulted in bias. In the MCAR and MAR scenario, multiple imputation including the outcome yielded the smallest mean squared errors, and combining multiple imputation and missing indicator method worked as efficient. In the MNAR scenario, combining multiple imputation and missing indicator method was slightly less biased than multiple imputation alone.

**Table 1:** Results of treatment effect estimates from propensity matching and propensity weighting when assuming there is a homogeneous treatment effect and no unmeasured confounding. For each missing scenario, missing data are handled with complete case analysis, missing indicator method, multiple imputation, and the combination of multiple imputation and missing indicator (Combined method)

		Homogeneous treatment effect					
		Propensity matching			Propensity weighting		
		Coefficient		MSE	Coefficient		MSE
		Mean	SD		Mean	SD	
No missing	No adjustment	1.298	0.123	1.700	1.298	0.123	1.700
	After adjustment	0.044	0.085	0.009	0.006	0.109	0.012
MCAR	Complete case analysis	0.043	0.121	0.016	0.014	0.152	0.023
	Missing indicator	0.238	0.095	0.066	0.189	0.111	0.048
	Multiple imputation						
	With Y	0.047	0.086	0.010	0.011	0.113	0.013
	Without Y	0.219	0.087	0.056	0.186	0.110	0.047
	Combined method						
	With Y	0.048	0.087	0.010	0.011	0.112	0.013
	Without Y	0.218	0.087	0.055	0.187	0.110	0.047
MAR	Complete case analysis	0.024	0.128	0.017	0.007	0.165	0.027
	Missing indicator	0.259	0.099	0.077	0.172	0.123	0.044
	Multiple imputation						
	With Y	0.052	0.092	0.011	0.010	0.122	0.015
	Without Y	0.244	0.090	0.068	0.185	0.120	0.049
	Combined method						
	With Y	0.050	0.092	0.011	0.010	0.122	0.015
	Without Y	0.243	0.090	0.067	0.185	0.120	0.048
MNAR	Complete case analysis	0.025	0.129	0.017	0.012	0.166	0.028
	Missing indicator	0.231	0.098	0.063	0.149	0.122	0.037
	Multiple imputation						
	With Y	0.069	0.095	0.014	0.029	0.123	0.016
	Without Y	0.248	0.091	0.070	0.215	0.118	0.060
	Combined method						
	With Y	0.052	0.093	0.011	0.011	0.122	0.015
	Without Y	0.211	0.088	0.053	0.160	0.119	0.040

### Simulation setting 2: No unmeasured confounding and a heterogeneous treatment effect

Figure 4 (middle) visualises the results of the propensity weighting analysis of Simulation setting 2, and Table 2 summarises the results of the propensity matching and the propensity weighting. Here the complete case analysis yielded negatively biased results in the MAR or MNAR scenarios. This is because the subjects with higher X_2_ values, for whom the treatment was most beneficial, had higher probabilities to be excluded in the analyses. The missing indicator method was still biased in all scenarios. The amount of bias, however, was relatively small in the MNAR scenario. We observed a remarkable result in the MAR scenario: the default multiple imputation method yielded biased effect estimates, even when the outcome was included in the imputation model and when a missing indicator was added to the propensity model. When more elaborate imputation regression models with specified interaction terms were used, the bias from the propensity weighting was much smaller, although a slight bias still remained (0.013).

**Table 2:** Results of treatment effect estimates from propensity matching and propensity weighting when assuming X_2_ is an effect modifier and no unmeasured confounder exists. Here, multiple imputation is done in two ways; commonly used method (no interaction term) and elaborated method (interaction terms included)

		Heterogeneous treatment effect						
		Propensity matching				Propensity weighting		
		Coefficient		Bias	MSE	Coefficient		MSE
		Mean	SD			Mean	SD	
No missing	No adjustment	1.736	0.156	1.409	2.011	1.736	0.156	3.040
	After adjustment	0.327	0.093	0.000	0.009	− 0.003	0.152	0.023
MCAR	Complete case analysis	0.300	0.133	− 0.027	0.018	− 0.003	0.219	0.048
	Missing indicator	0.574	0.120	0.247	0.075	0.305	0.162	0.119
	*No interaction term*							
	Multiple imputation							
	With Y	0.315	0.103	− 0.012	0.011	− 0.021	0.168	0.029
	Without Y	0.542	0.108	0.215	0.058	0.297	0.158	0.113
	Combined method							
	With Y	0.315	0.102	− 0.012	0.011	− 0.021	0.169	0.029
	Without Y	0.541	0.110	0.214	0.058	0.297	0.158	0.113
	Interaction terms							
	Multiple imputation	0.316	0.103	− 0.011	0.011	− 0.002	0.166	0.028
	Combined method	0.316	0.104	− 0.011	0.011	− 0.003	0.166	0.028
MAR	Complete case analysis	0.129	0.147	− 0.198	0.061	− 0.200	0.241	0.098
	Missing indicator	0.620	0.122	0.293	0.101	0.272	0.179	0.106
	*No interaction term*							
	Multiple imputation							
	With Y	0.251	0.107	− 0.076	0.017	− 0.093	0.181	0.042
	Without Y	0.579	0.112	0.252	0.076	0.286	0.173	0.111
	Combined method							
	With Y	0.250	0.108	− 0.077	0.017	− 0.092	0.182	0.042
	Without Y	0.580	0.113	0.253	0.077	0.285	0.173	0.111
	Interaction terms							
	Multiple imputation	0.330	0.116	0.003	0.013	0.010	0.185	0.034
	Combined method	0.330	0.116	0.003	0.013	0.010	0.185	0.034
MNAR	Complete case analysis	− 0.111	0.141	− 0.438	0.211	− 0.411	0.224	0.219
	Missing indicator	0.588	0.121	0.261	0.082	0.230	0.171	0.082
	*No interaction term*							
	Multiple imputation							
	With Y	0.151	0.114	− 0.176	0.044	− 0.238	0.207	0.100
	Without Y	0.586	0.112	0.259	0.080	0.350	0.165	0.150
	Combined method							
	With Y	0.140	0.111	− 0.187	0.047	− 0.248	0.206	0.104
	Without Y	0.546	0.108	0.219	0.060	0.248	0.165	0.089
	Interaction terms							
	Multiple imputation	0.182	0.117	− 0.145	0.035	− 0.192	0.208	0.080
	Combined method	0.170	0.114	− 0.157	0.038	− 0.205	0.264	0.112

The results of propensity matching even in the situation without any missing values (0.327) deviated from the treatment effect in all treated subjects (0.432). This discrepancy is a general problem of propensity score matching [37–39]. A large caliper distance allows treated subjects with high propensity scores to be matched to untreated subjects with lower propensity scores, which will result in residual confounding. A smaller caliper distance reduces the confounding bias. However, many subjects, especially the subjects with high propensity score, may not be matched. Therefore, the treatment effect in the treated *who are matched* may deviate from the treatment effect in *all* treated. The size of this discrepancy depends on the heterogeneity of the treatment effect. In this simulation setting, we used matching without replacement with a caliper distance of 0.1, which allows rather tight matching. Thus, for some of the treated subjects with high propensity score, whose treatment effect was more effective, no adequate untreated match could be found. As we were specifically interested in the additional bias under the different missing mechanisms, we used the estimate of propensity matching without any missing data (0.327) as a reference. Once more, we observed that multiple imputation with interaction terms performed best as it did in propensity weighting analysis.

The results of multiple imputation with Bayesian regression methods done in a sensitivity analysis did not largely differ from the results of predictive mean matching (see Appendix 1, for the results in Simulation setting 2).

### Simulation setting 3: Unmeasured confounding and a homogeneous treatment effect

Figure 4 (right) displays the results of the propensity weighting of Simulation setting 3, and Table 3 summarises the results of propensity matching and propensity weighting. Due to the unmeasured confounder U, bias remained in the propensity analyses even when there were no missing values. In the MNAR scenario where the missingness of X_2_ depends on U, two methods were able to reduce the unmeasured confounding effect: the combined method and, somewhat surprising, the complete case analysis. The combined method partially adjusted for U by adding R to the propensity model; the complete case analysis used restriction to partially adjust for U, using only those with complete data. The results here were substantially less biased than the propensity analyses performed in complete data without missing values.

**Table 3:** Results of treatment effect estimates from propensity matching and inverse probability weighting, when an unmeasured confounding exists

		Homogeneous treatment effect/unmeasured confounding					
		Propensity matching			Propensity weighting		
		Coefficient		MSE	Coefficient		MSE
		Mean	SD		Mean	SD	
No missing	No adjustment	2.011	0.154	4.068	2.011	0.154	4.068
	After adjustment	0.377	0.111	0.154	0.328	0.168	0.136
MCAR	Complete case analysis	0.362	0.152	0.154	0.336	0.233	0.167
	Missing indicator	0.870	0.138	0.776	0.774	0.171	0.628
	Multiple imputation						
	With Y	0.376	0.119	0.155	0.330	0.171	0.138
	Without Y	0.807	0.119	0.665	0.771	0.165	0.621
	Combined method						
	With Y	0.375	0.119	0.155	0.330	0.171	0.138
	Without Y	0.808	0.119	0.667	0.770	0.165	0.620
MNAR	Complete case analysis	0.145	0.163	0.048	0.157	0.255	0.089
	Missing indicator	0.514	0.117	0.277	0.345	0.197	0.158
	Multiple imputation						
	With Y	0.422	0.141	0.197	0.354	0.200	0.165
	Without Y	1.003	0.129	1.023	1.028	0.154	1.079
	Combined method						
	With Y	0.240	0.114	0.071	0.169	0.191	0.065
	Without Y	0.469	0.105	0.231	0.386	0.175	0.180

## Guidance for the optimal strategy to handle missing values in baseline covariates in the context of propensity score analysis

The aim of a propensity score analysis is to obtain an average treatment effect in a certain population. To explain, we use the following notation in which every subject can have two potential outcomes:

- Y^1^; the outcome if the person receives treatment 1.
- Y^0^; the outcome if the person receives treatment 0.

Propensity weighting aims to estimate the average treatment effect in the *whole population* (ATE), which is equal to: $ATE = E\left[ {Y^{1} - Y^{0} } \right].$ With propensity matching, where treated subjects are matched to untreated subjects, the aim is to estimate the average treatment effect in the *treated population* (ATT): $ATT = E\left[ {Y^{1} - Y^{0} |{\text{T}} = 1} \right].$ Several standard causal inference conditions such as exchangeability, consistency and positivity should hold to be able to estimate these causal effects without bias [40]. Whether the unbiased causal effects can still be estimated when missing values are present in the covariates of a propensity score depends on several elements: type of missingness, presence of effect modification and the population of interest. In the following section, we discuss under which criteria the four methods dealing with missing values will yield valid causal treatment effect in the context of propensity score analysis.

### Complete case analysis, when does it work?

When there is no unmeasured confounding and the propensity score model is well specified, propensity weighting using complete cases will yield a valid estimate of a causal treatment effect, which will be the causal treatment effect in the *subjects without missing values:*

$$E\left[ {Y^{1} - Y^{0} | R = 0} \right]$$

This means that propensity weighting using complete case analysis will yield valid estimates of the ATE in the population when the mean treatment effect in the fully observed subjects is equal to that of the subjects with missing values. That is:

1 $$\begin{array}{*{20}c} {E\left[ {Y^{1} - Y^{0} | R = 0} \right] = E\left[ {Y^{1} - Y^{0} | R = 1} \right] = E\left[ {Y^{1} - Y^{0} } \right] } \\ \end{array}$$

When data are missing completely at random, condition (1) will hold, because the probability of a missing value does not depend on any observed or unobserved variable. This means that the covariate with missing values is independent of its own missing indicator variable. The m-graphs may be helpful in identifying whether this independency holds. In the m-graphs in Figs. 1a and 2a, these conditions hold because X_2_ and R are unconditionally *d*-*separated*, meaning that there is no open path between X_2_ and R.

When no effect modification and no unmeasured confounding is present, condition (1) will also hold since the treatment effect in the total population will be equal to the treatment effect in any subgroup regardless of the missing mechanism of data. This was the case in Simulation 1 where the effect of the treatment was constant across subjects. In this scenario, the complete case analysis yielded unbiased results even when the missing was not at random. Analogous arguments can be given for propensity matching using complete cases. The propensity matching will yield valid estimates if:

2 $$\begin{array}{*{20}c} {E\left[ {Y^{1} - Y^{0} | R = 0, T = 1} \right] = E\left[ {Y^{1} - Y^{0} | R = 1, T = 1} \right] = E[Y^{1} - Y^{0} |T = 1]} \\ \end{array}$$

Even when there is unmeasured confounding, complete case analysis may be a useful way to handle missing values. Think of a situation where the severity of a disease determines whether certain laboratory tests will be performed. Severity of disease here may be an unmeasured confounder, which determines the values of observed covariates (in this case the laboratory measurements) to be missing. This is a comparable situation to the MNAR scenario of Simulation setting 3. Here, the complete case analysis yielded less biased results, because by restricting the analysis to subjects with R = 0 (only the subjects with the severe diseases who therefore have all lab measurements), the results are partially adjusted for the unmeasured confounder.

### Missing indicator, when does it work?

In general, we do not recommend to solely use the missing indicator method for handling missing values in confounders. The method is prone to result in bias because the information of the missing portion of the covariates is replaced by a dichotomous missing indicator R, consequently resulting in residual confounding. However, when data are missing not at random and the covariate with missing value is strongly associated to its missing indicator, the missing indicator variable in a propensity model may yield smaller bias than the model without it. This was the case in the MNAR scenarios of Simulation setting 1 and 2. Similarly, when the missing of X_2_ is strongly related to an unmeasured confounder U, the partial effect of U can be recovered by adding R in the propensity model. This was seen in the MNAR scenario of Simulation 3.

### Multiple imputation, when does it work?

The aim of multiple imputation is to recover the joint distribution of covariates, treatment and outcome by reconstructing the missing values using the information from observed data. When there is no unmeasured confounding, multiple imputation in the context of propensity score analysis will be a valid approach under the following conditions:

1. Data are missing at random or completely at random, meaning that the missing values are *recoverable* from the observed data. M-graphs can be used to visually determine whether the missing mechanism is at random. In m-graphs, the missing at random mechanism means that all paths between a covariate with missing values and its missing indicator can be blocked by conditioning on measured variables. In DAG terms, it is said; two variables are *d*-*separated*. In our study, this was the case in Figs. 1a, b and 2a, b. Note that in Figs. 1b and 2b, the path between X_2_ and R can be blocked by conditioning on X_1_.
2. An imputation model should be correctly specified. This requires that:
  - the outcome should be included in the imputation model.
  - interaction terms between the covariates, treatment and outcome should be included in the imputation model if a heterogeneous treatment effect is present.

In Simulation setting 1, multiple imputation yielded unbiased results even though it was used to impute a non-recoverable X_2_. Note that the reason why multiple imputation worked well in this scenario was because 1) the covariates, treatment and the outcome in the model were linearly related, and 2) missing values in X_2_ were generated probabilistically which means the information of higher X_2_ values could be gained from the data. This result is due to the simulation scenario we generated and should not be taken to show that multiple imputation can be used when data are missing not at random.

### What to do in situations where complete case analysis or multiple imputation fails?

We saw in the previous section it is important that a researcher is aware of the missing mechanism and whether strong heterogeneity is present. Depending on the missing mechanism and the heterogeneity in the treatment effect, both complete case analysis and multiple imputation may fail. Whether the treatment effect is heterogeneous can be explored by subgroup analysis and comparing the estimated effects across the groups. When there is large difference across the subgroups, interaction terms should be specified in the multiple imputation. This was shown in Simulation setting 2.

The missing mechanism behind the data can be explored by drawing the expected causal structure and missing structure in a m-graph. When complete case analysis and multiple imputation are expected to fail, the combination of multiple imputation and the missing indicator method could be used to partially recover the effect of missing portions of covariates. For example, in the MNAR scenario of Simulation setting 3, the combined approach performed better than multiple imputation alone and even better than the analysis on the data without any missing values. When the relation between R and U is stronger, more of the effect of the unmeasured confounder will be recovered.

## Discussion

Our simulations showed that there is no single method to handle missing values in covariates of a propensity score model which would perform optimally in all situations. The optimal strategy depends on the missing data structure and whether there is effect modification or unmeasured confounding. We focussed on missing values in covariates, because in routinely collected data baseline patient characteristics are often incomplete while prescribed treatments and important outcomes of patients will be more generally recorded.

Our results cannot be generalized to the situations when there are missing values in the treatment assignment or the outcome. An example of this is that under homogenous treatment effect and no unmeasured confounding, complete case analysis will yield biased results if the outcome is missing not at random.

Propensity score analysis mimics randomized control studies by creating conditional exchangeability between the subjects with the same propensity score. Both propensity weighting and matching aim to obtain valid estimates of marginal treatment effects. This is different from outcome regression analysis which estimates conditional treatment effects. Unlike outcome regression model, no assumptions about treatment-outcome relation and the effect of the confounders on the outcome have to be made in propensity score analysis; only the propensity score model has to be correctly specified. This is an advantage, especially when the outcome is rare in which case fitting an extensive outcome model is not possible.

When using multiple imputation, the advantage of not having to formulate a treatment-outcome relation model disappears. In our simulations we showed that all variables associated with the covariates with missing values, including the outcome, should be included in the imputation model. Furthermore, when effect modification is present, the interaction terms between the variables should be correctly specified in the imputation model as well. The results correspond to the idea that imputation models should reflect the complexity of the data analysis procedure [41, 42]. When complex modelling is needed for multiple imputation, an alternative to propensity score analysis could be to use an outcome regression model with specified interaction terms. By fitting this outcome regression model, one can predict potential outcomes under treatment and no treatment for *every individual*. Then, the *average* potential outcomes can be estimated by integrating over the covariate distribution, and used to obtain the average treatment effect in the population [40].

Multiple imputation is not a panacea to handle missing values, and should be used more consciously. In our simulations we demonstrated that a default option for multiple imputation in commonly used software such as SAS, Stata, SPSS or R yielded biased results (based on Simulation setting 2) even when data were missing at random and no unmeasured confounding was present.

Complete case analysis may often be a good method to deal with missing values in covariates. Although statistical efficiency is lost, estimated effects still have a causal interpretation if there is no unmeasured confounding. In these cases, it is up to the researcher to determine how generalizable these results are to the general population of interest. In the case of substantial heterogeneity of treatment effects, generalizability cannot be taken for granted.

When unmeasured confounding is present, all standard missing data methods fail to provide valid estimates. Complete case analysis, however, may reduce the bias by controlling the unmeasured confounding by restriction. The use of an indicator variable (with or without multiple imputation) may also reduce the bias, because the indicator variable functions as a proxy for the unmeasured confounding.

A recent systematic review on how missing data are addressed with propensity score methods in observational comparative effectiveness studies showed that among 167 studies conducted from 2010 to 2017, only 86 (51%) discussed missing data issues and only 12 (7%) provided reasons for missingnesss [43]. Our simulation study showed that it is important to make assumptions about the expected relationship between the unobserved and observed covariates. This allows one to understand the expected missing structure of the data and to handle missing values more cautiously. We recommend researchers to use m-graphs to draw their assumption between the covariates and their missing indicator explicitly. In summary, in the context of propensity score analysis we urge researchers to consciously choose missing data strategies while considering the missing data mechanisms, possible unmeasured confounding and heterogeneity of treatment effects.

## Appendix

See Table 4.

**Table 4:** Results of Simulation setting 2 where the multiple imputation by chained equations (MICE) with Bayesian linear regression is used  for a sensitivity analysis

	Heterogeneous treatment effect						
	Propensity matching				Propensity weighting		
	Coefficient		Bias	MSE	Coefficient		MSE
	Mean	SD			Mean	SD	
No adjustment	1.730	0.158	1.410	2.012	1.730	0.158	3.019
After adjustment	0.321	0.096	0.000	0.009	− 0.017	0.156	0.025
*No interaction term*							
Multiple imputation							
With Y	0.304	0.095	− 0.017	0.009	− 0.041	0.170	0.031
Without Y	0.536	0.101	0.215	0.056	0.292	0.142	0.105
Combined method							
With Y	0.303	0.095	− 0.018	0.009	− 0.042	0.172	0.031
Without Y	0.537	0.104	0.216	0.058	0.294	0.143	0.107
Interaction terms							
Multiple imputation	0.315	0.094	− 0.006	0.009	− 0.014	0.169	0.029
Combined method	0.315	0.096	− 0.006	0.009	− 0.015	0.171	0.029
*No interaction term*							
Multiple imputation							
With Y	0.220	0.103	− 0.101	0.021	− 0.116	0.192	0.050
Without Y	0.568	0.110	0.247	0.073	0.264	0.158	0.095
Combined method							
With Y	0.220	0.101		0.010	− 0.116	0.190	0.049
Without Y	0.568	0.111	0.248	0.074	0.264	0.157	0.094
Interaction terms							
Multiple imputation	0.330	0.101	0.009	0.010	0.002	0.199	0.040
Combined method	0.331	0.103	0.010	0.011	0.001	0.198	0.039
*No interaction term*							
Multiple imputation							
With Y	0.102	0.110	− 0.219	0.060	− 0.269	0.213	0.118
Without Y	0.570	0.110	0.249	0.074	0.325	0.153	0.129
Combined method							
With Y	0.095	0.103	− 0.225	0.061	− 0.275	0.211	0.120
Without Y	0.537	0.105	0.216	0.058	0.233	0.149	0.076
Interaction terms							
Multiple imputation	0.173	0.101	− 0.147	0.032	− 0.197	0.220	0.087
Combined method	0.169	0.103	− 0.151	0.034	− 0.206	0.215	0.089
